# Dynamic Group Authentication and Key Exchange Scheme Based on Threshold Secret Sharing for IoT Smart Metering Environments

**DOI:** 10.3390/s18103534

**Published:** 2018-10-19

**Authors:** Dae-Hwi Lee, Im-Yeong Lee

**Affiliations:** Department of Computer Science and Engineering, Soonchunhyang University, Asan 31538, Korea; leedh527@sch.ac.kr

**Keywords:** IoT, smart metering environments, group authentication, key exchange, threshold secret sharing

## Abstract

The Internet of Things (IoT) environment is constantly evolving. Many IoT services have emerged, improving living conditions. Smart homes were among the first developments, and smart buildings, smart factories, and smart cities are attracting increasing attention. Smart cities represent the ultimate convergence of the IoT, the Cloud, big data, and mobile technology. Smart homes, buildings, and factories create smart cities. In addition, the IoT finds applications in traffic control, public safety, and medical services, permitting group-based communication. As the scale of service grows, the number of things (devices) constituting the service also increases. However, security vulnerabilities arise in group-based communication environments. A device may require authentication when entering a gateway; to secure environments with large numbers of devices (such as those featuring IoT smart metering), the gateways bear heavy loads. Therefore, efficient authentication of group leaders and devices is essential. Here, we develop a dynamic group authentication and key exchange scheme for group-based IoT smart metering environments which enables efficient communication among secure IoT services. Our group authentication scheme increases the computational efficiency of the group leader and the participating devices, based on a threshold secret sharing technique.

## 1. Introduction

**Figure 1:**
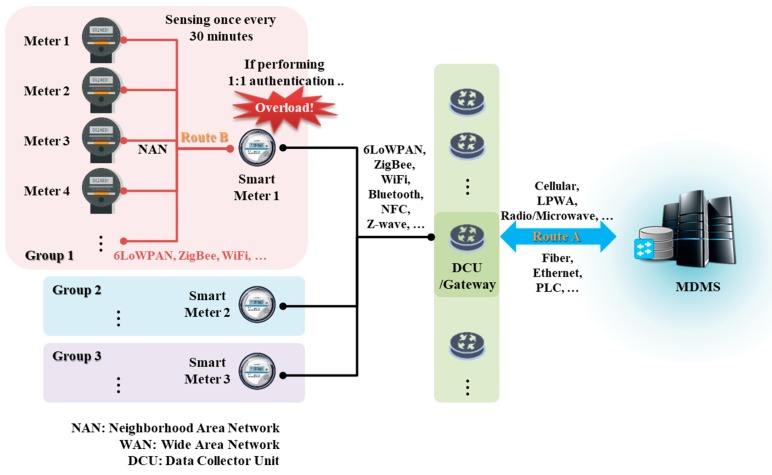
Problems encountered when performing one-to-one authentication in a smart metering environment.

The Internet of Things (IoT) is an environment within existing wireless sensor networks (WSNs), wherein all devices are connected to the Internet, for data collection and to provide services based on such data. Recently, various convenient IoT-based services have been developed, commencing with small services such as Wireless Body Area Networks (WBANs) and progressing to smart homes, buildings, factories, and cities [1]. Particularly, as the fourth industrial revolution proceeds, research on, and service development of, the IoT, Cloud, big data, and mobile technology (ICBM) cluster is progressing rapidly, aiming toward smart cities [2]. In various IoT service environments characteristic of smart cities, many sensors, users, and services connect to share data. Of these devices, gateway- and server-class devices can afford good computing power, but general IoT sensor devices are of very low power. Therefore, a security protocol, such as authentication, that operates within sensor devices must be lightweight. As the sizes of communication networks increase, IoT services engage in group-based communication [3]. Various sensor devices collect data in a group format, and then transmit them to a group leader serving as a gateway. For example, in a smart metering environment, IoT devices and home appliances form a home group and send power consumption data to a smart meter (a group leader). If the leader communicates with the devices, power consumption data are collected via one-to-one communication and no group is formed. Such communication is possible when the number of devices is small, but as the number of devices increases, problems arise. Figure 1 shows the communication flow of smart meters in a smart metering environment for the process of collecting power consumption data. There is a lot of overhead in performing a one-to-one authentication on a smart meter. The group leader must authenticate many devices and engage in secure communication only after key exchange.

A smart home can serve as a group that communicates with a service provider (which updates that group), or with a neighboring group, or a single user-device group, in a WBAN environments. However, if the number of devices in a group increases, or when many devices must communicate simultaneously, authentication of all devices is required during each session to ensure that communication is secure. Highly efficient group authentication technology is thus required.

Here, we develop a dynamic group authentication and key exchange scheme that operates efficiently in lightweight devices within a group-based IoT smart metering environment. As the number of devices in the group increases, the number of communications in the group leader traditionally becomes very large. Therefore, we structure our scheme to ensure that the group leader engages with an authentication server to ensure key agreement. Also, we develop a key exchange process generating and distributing group keys after group authentication, via a threshold secret sharing scheme; this reduces the group communication overhead.

## 2. Related Work

Here, we initially discuss prior research on group authentication and key exchange techniques for IoT smart metering environments. First, we discuss how group communication is achieved in such environments, and the threshold secret sharing techniques used for group authentication. Then, we describe our group authentication technique and analyze existing schemes.

### 2.1. Smart Metering Environments

**Figure 2:**
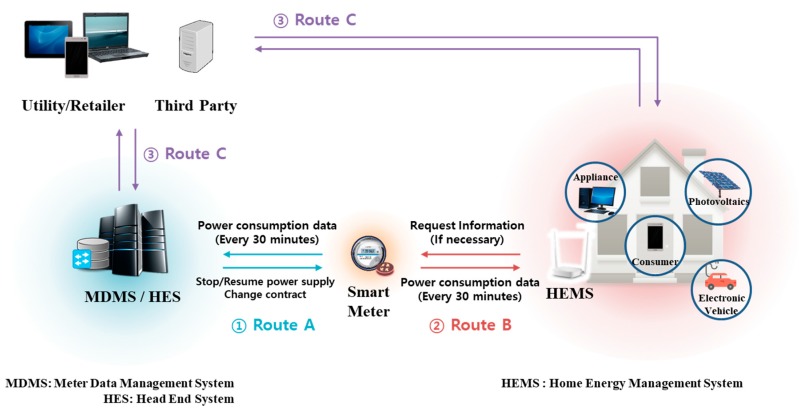
Three communication paths in a smart metering environment.

Smart metering is an IoT service that remotely records home power consumption [4,5]. Figure 2 shows the communication routes for each object. The smart meter is a group leader that collects power consumption data on home appliances and transmits them to a home energy management system (HEMS). In a smart metering environment, communication paths A, B, and C are active [6]. Path A is that from the smart meter to the power company; the meter transmits data to the company’s meter data management system (MDMS), or the MDMS uses power consumption data to pause/renew or change the power contract. Path B is that through which the HEMS and smart meter communicate, used when the HEMS sends data from the home to the smart meter or when the customer wants to access smart meter data or check power consumption. Finally, Path C is used by third parties (such as energy service companies) that require smart meter information.

When a smart meter reads HEMS power consumption data using Path B, the meter will simultaneously engage in one-to-one authentication of a large number of devices online. As the HEMS services a group of general or smart homes, group authentication would allow the smart meter to operate more efficiently. Also, to securely transmit power consumption data collected via route B to the MDMS, the smart meter and the MDMS must engage in mutual authentication of transmit/receive actions using a dedicated session key. Smart meters can also form groups, but as each smart meter manages data of different groups, and as group attributes differ, mutual authentication of the smart meter and the MDMS is preferable.

### 2.2. Smart Metering Environments Security Threat Model

In IoT environments, security technologies are developed in terms of authentication and privacy preserving. In a smart metering environment, the main security issues concern authentication and privacy problems. Various schemes have been proposed to solve authentication and privacy problems in the process of collecting and transmitting power consumption data through smart meters [7,8,9,10]. This section describes some security threat models in smart metering environments.

The IoT smart metering environment operates as described in Section 2.1. In terms of efficiency in authentication, smart meters perform group authentication with MDMS more efficiently than existing smart metering services. Group authentication is discussed in Section 2.3 and this section discusses security threats in this environment.

In path B in Figure 1, the smart meter checks the power consumption data of the HEMS in a smart home. If authentication is not performed properly in this process, a risk of replay attacks occurs. If an attacker’s unauthorized smart meter could request and receive power consumption data from the HEMS, there would be a privacy problem in that the attacker would be able to identify the power consumption data of the smart home [7,8]. In addition, power consumption data of the forged HEMS may be transmitted to the smart meter and stored in the MDMS, thereby causing the problem of performing an incorrect meter reading. Likewise, in path A, there is a problem in that the forged power consumption data of a certain assumption can be transmitted to the MDMS. To prevent this problem, authentication of each communication participating object is required [8,9,10].

It is desirable to securely communicate by obtaining the session key after mutual authentication of the object. Aman-in-the-middle attack in the process of performing the authentication should also be considered. If an attacker performs a replay attack by taking a packet that communicates with the smart meter, HEMS, or MDMS at the time of performing the authentication, the damage described above may occur. Therefore, the communication protocol must be designed to be secure against a replay attack.

### 2.3. Threshold Secret Sharing

**Figure 3:**
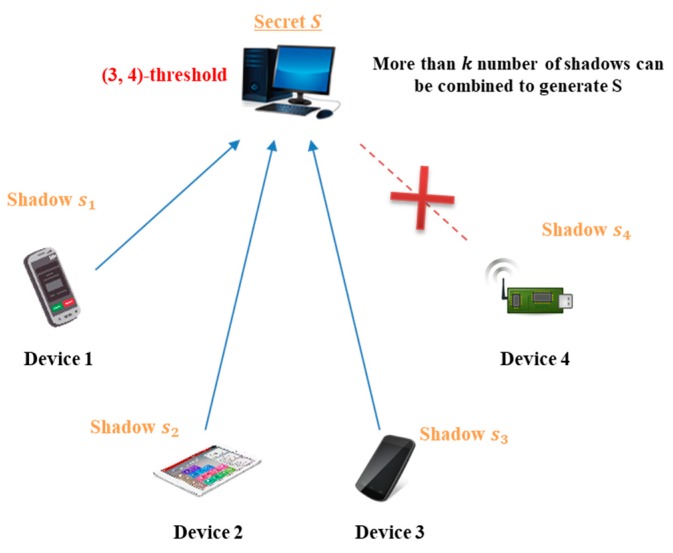
Structure of (3,4)-threshold secret sharing.

The secret sharing technique has long been used to manage confidential information, such as keys. The technique encrypts and stores keys used to decrypt encrypted protected information [11]. If the key is lost or the person managing the key dies, the encrypted information cannot be decrypted and, when only a key is used, may be leaked. To prevent this, division and storage of secret information, such as that on a decryption key, is required. Common secret sharing schemes feature the (*t*, *n*)-threshold secret sharing scheme of Shamir [11]. The scheme can be restored only if *t* of the *n* participants sharing secret information are involved in such restoration. Figure 3 shows (3,4)-threshold secret sharing. If three of the four devices participate, the secret, S, can be recovered. The scheme features polynomials within a finite field and restores keys between polynomials via Lagrangian interpolation. Other secret sharing techniques feature different polynomials, or the use of various mathematical principles such as Exclusive-OR (XOR) [12,13,14], Chinese remainder theorem (CRT) [15,16], or hash functions [17,18].

### 2.4. Group Authenticaion

**Figure 4:**
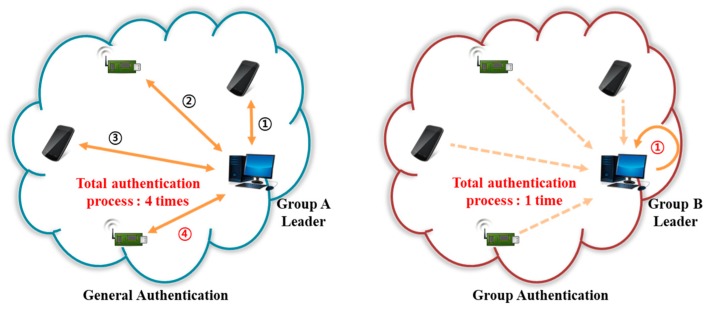
Comparison of general single authentication and group authentication.

In an IoT network, many users and devices are distributed in a non-centralized manner, which causes issues in terms of authentication, access control, and identification within a distributed environment. Security challenges, heterogeneous communications, and resource constraints must be considered. In particular, authentication and identification management are very important and must be simple, safe, and fast. In a distributed environment, several devices constitute a group unit and secure group communication is essential [3]. A group authentication technique determines whether a user, or a device of a group participant, belongs to that group, and performs authentications in an environment in which the group leader communicates with devices in a one-to-one manner. Such technology secures communication by requiring only a single authentication when group members seek to communicate. Figure 4 compares general single authentication and group authentication. When four devices authenticate with the group leader with general single authentication, the authentication process is executed four times. On the other hand, with group authentication, the authentication process performed by the group leader is executed only once. The various schemes are described as intra- and inter-group authentications [3]. 

**Figure 5:**
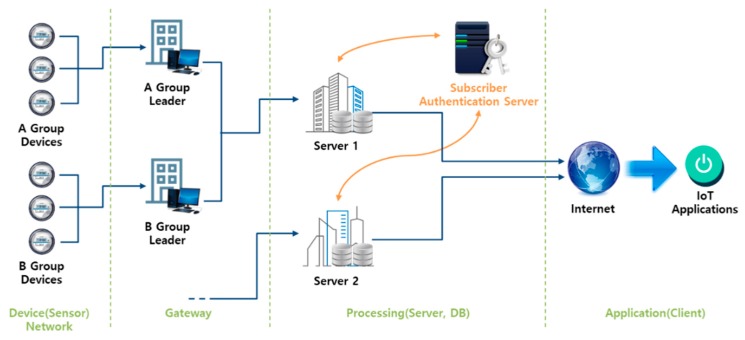
Group authentication scheme structure with an authentication server.

Group authentication is achieved principally by means of an authentication server, typically running the extensible authentication protocol (EAP) of the IEEE 802.1x standard, appropriate for ad hoc wireless networks and mobile users [19,20,21,22]. Figure 5 shows the communication flow applied in an IoT environment, such as smart metering, by a group authentication scheme with an authentication server.

**Figure 6:**
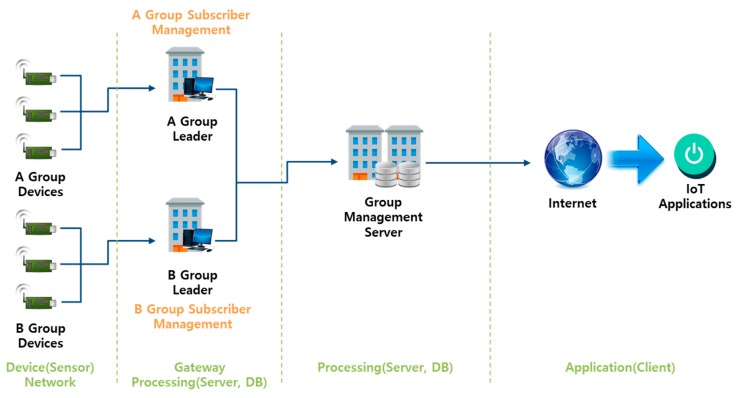
Group authentication scheme (GAS) structure without an authentication server.

In a scheme without an authentication server, a group leader is generally identified; this leader monitors when devices join and leave the group [23,24,25,26,27,28,29]. Earlier group authentication schemes (GASs) were based on a “threshold secret sharing” technique. Group authentication not only manages group participants, but also performs hierarchical group-based authentication using a group key. Figure 6 shows the communication flow applied in an IoT environment such as smart metering, by Harn’s group authentication scheme (GAS) with the authentication server.

The GAS of Harn (2013) uses the Shamir-threshold secret sharing scheme [23]. If *m* users (exceeding the threshold of *t* users among *n* users) participate, group authentication is successful. Harn proposed a new branched scheme for asynchronous (*t*, *m*, *n*) group authentication (GAS1). However, in GAS1, once a secret is used for authentication, it is no longer secret; all secrets are disposable and cannot be re-used. Asynchronous (*t*, *m*, *n*) group authentication with multiple authentication (GAS2) is a group authentication technique not affected by this problem. However, with GAS2, it is possible to collect public tokens to access the secret, and then to use the secret to determine the secret values of other participants for a spoofing attack [25].

To solve this GAS2 problem, Chien (2017) created secret values using tokens published with the aid of the elliptic curve discrete logarithm problem [25]. Although the tokens are disclosed using an arbitrary point on an elliptic curve determined before each group authentication session, redistribution is required when all points are both distributed and used. In addition, between-node synchronization is required using a previously disclosed value when an internal group node does not participate in group authentication. Furthermore, the Harn and Chien schemes broadcast tokens to nodes participating in group authentication during authentication. This process (resembling an IoT environment) is hierarchical and does not ensure secure communication in an environment in which communications are connected and a group leader manages devices. We solve this problem by devising a GAS that operates safely in IoT communication environments but employs reusable authentication.

## 3. Security Requirements

Here, we analyze the security requirements of efficient group authentication and key exchange schemes for IoT smart metering environments. In an IoT environment in which many devices are connected, there should be no limit on the number of group members; thus, a management scheme is imperative. Also, as described in Section 2.2, various threats can occur in a smart metering environment, and the computational overhead must be low to prevent replay attacks and allow adaptation to the environment [26,27]. We propose that each device should be authenticated to safely read power consumption data.

### 3.1. Authentication

Each entity within a group must prove that it is participating legitimately via the meter nodes, smart meters and MDMS. During group authentication, the group leader manages all participants, who are not individually authenticated; the group leader performs all authentications simultaneously.

### 3.2. Prevention of Replay Attacks

If the intermediate values used for authentication and key exchange are revealed to an attacker, that attacker should not be authenticated as a legitimate user when s/he retransmits the value to the group leader. In our threshold-based proposal scheme, even if the token intermediate value is exposed, the secret value, distributed secret value, and polynomial cannot be calculated. If the existing Harn scheme is used, secrets are disposable and spoofing attacks are possible.

### 3.3. Efficiency

During one-to-one mutual authentication, the larger the group size, the greater the communication overhead of the group leader. Therefore, group authentication is essential to improve group leader efficiency in terms of both authentication and key exchange. Group participants with lightweight nodes must not be asked to perform complicated operations and any such computations involving the group leader must be minimized.

### 3.4. Identifying Malicious Participants

When a group is authenticated, a malicious participant must not be able to engage in authentication. To this end, as in the existing Harn scheme, information created using an identifier must be verified during authentication. Although the Chien scheme solves this problem, it remains difficult to identify malicious group leaders, although malicious participants can be found because their communication structures differ. Therefore, during group authentication, it is necessary to use identifiers to find malicious participants and prohibit their participation.

## 4. Proposed Scheme

**Figure 7:**
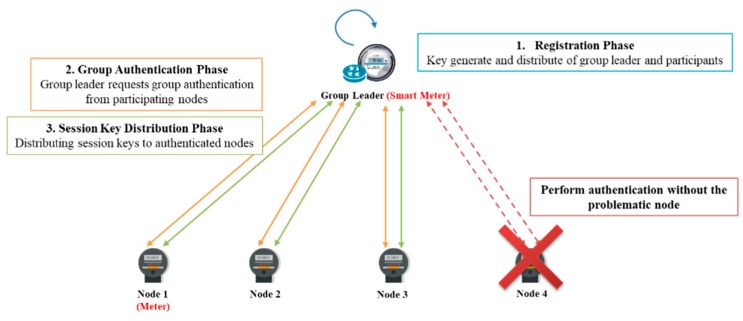
The smart meter intra-group authentication scheme.

**Figure 8:**
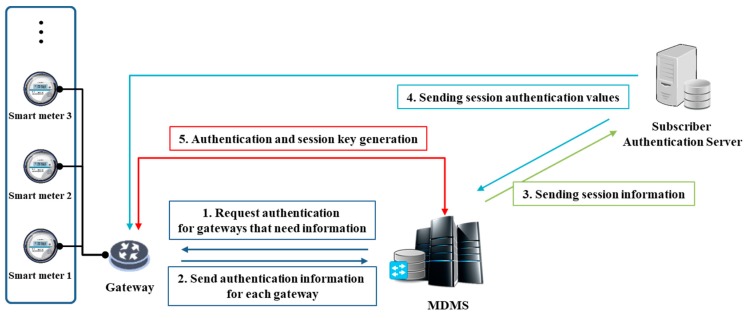
The meter data management system (MDMS)-gateway authentication phase.

Here, we develop a threshold-based dynamic group authentication and key exchange scheme for an IoT smart metering environment. The scheme is a smart meter intra-group scheme, which can later be extended to an MDMS-gateway group authentication scheme. As shown in Figure 7, the smart meter intra-group authentication scheme is divided into a node registration phase, a nodes-smart meter group authentication phase, and a group session key distribution phase. In addition, the MDMS-gateway group authentication is shown in Figure 8. The system parameters of the proposed scheme are shown in Table 1. If a meter node in the nodes-smart meter group authentication phase is not functioning properly, group authentication can be performed except for this meter node, which is identifiable by the smart meter. Thereafter, an action can be taken on the failure of the corresponding meter node in the MDMS.

### 4.1. Node Registration Phase

In the node registration phase, the meter nodes are newly registered by the smart meter. The smart meter first generates a single group master key, uses it to generate a secret key for participating meter nodes, and then employs it to distribute a session key. Secret values are securely distributed to nodes using a threshold secret sharing scheme.

Step 1. The smart meter generates a master key X.

Step 2. Participating meter nodes request the smart meter to join them.

Step 3. The smart meter generates a t−1 degree polynomial f(x) and computes a secret value sc.

(1) $$f\left(x\right)=a_{0}+a_{1}x+a_{2}x^{2}+\ldots +a_{t-1}x^{t-1}\;\;\;\;\;\left(a_{0}=s,\;\left[a_{1},\ldots ,a_{n}\right]\in Z_{p}^{*}\right)$$

Step 4. The smart meter calculates a meter node secret key $b_{m_{i}}=h\left(X∥ID_{m_{i}}\right)$ and a distributed secret value $f\left(ID_{m_{i}}\right)$ of each meter node.

Step 5. The smart meter calculates and discloses a verification value $h\left(scG_{r}\right)$ of the secret value sc.

Step 6. The smart meter sends $f\left(ID_{m_{i}}∥b_{m_{i}}\right)$ to each node i over a secure channel, and the nodes store the received value. 

### 4.2. Nodes-Smart Meter Group Authentication Phase

In the nodes-smart meter group authentication phase, the group leader requests the group to accept data from participating meter nodes, and simultaneously confirms and authenticates participation. Here, the smart meter checks all nodes for maliciousness, the meter nodes generate authentication tokens using the secret values received at the registration phase, and these tokens are reusable. The meter nodes generate tokens and send them to the smart meter, which confirms the nodal group. When transmitting tokens, replay attacks must be considered and it must be impossible to recover the value of a secret polynomial even if the token is collected. The smart meter receives all tokens and then engages in group authentication using other tokens.

Step 1. The smart meter broadcasts a group authentication request to participating meter nodes.

Step 2. The participating meter nodes check the request and send an m-node set $\left\{P_{1},P_{2},\ldots ,P_{m}\right\}$ to the group leader.

Step 3. The smart meter sends an arbitrary point $P_{s}$ on the elliptic curve, the number, *m,* of participating meter nodes, and a randomly selected hash function $h_{r}\left(\cdot \right)$, and requests a token from each node.

Step 4. Each meter node calculates a $c_{i}$ value using a distributed secret value, as follows:

(2) $$c_{i}=\overset{m}{\underset{r=1,r\neq i}{\prod }}\frac{-ID_{m_{r}}}{ID_{m_{i}}-ID_{m_{r}}}\;\left(mod\;p\right)$$

Step 5. The meter nodes use their computed $c_{i}$ values to calculate their tokens $T_{i}$, as shown below. Thereafter, a verification value $v_{i}$ is generated.

(3) $$T_{i}=c_{i}P_{s},\;v_{i}=h_{r}\left(b_{m_{i}}∥T_{i}\right)$$

Step 6. Authenticated meter nodes $\left\{P_{1},P_{2},\ldots ,P_{m}\right\}$ transmit individual $\left(ID_{m_{i}}∥v_{i}∥T_{i}\right)$ blocks to the smart meter.

Step 7. The smart meter calculates new verification values, $v_{i}$, for each node, and then calculates V as follows:

(4) $$V=\overset{m}{\underset{r=1}{\sum }}T_{r}\;\left(mod\;p\right)$$

Step 8. The smart meter verifies matching of $h_{r}\left(V\right)=h_{r}\left(scG_{r}\right)$ using the secret value sc, and authenticates *m* participating meter nodes. If the above verification is not matched, group authentication fails.

### 4.3. Group Session Key Distribution Phase

In the session key distribution phase, a key generated by the smart meter is encrypted using the secret key for each meter node and then distributed. Although meter nodes can generate individual session keys using the Harn authentication scheme, the group leader reduces node operation overheads by performing that role. However, the group leader, such as the smart meter, must encrypt each session key with a different secret key, associated with a high computational overhead.

Step 1. The smart meter generates a group session key $GSK=h_{s}\left(P_{s}∥X∥sc\right)$, and a $\left\{P_{1},P_{2},\ldots ,P_{m}\right\}$ encrypting that key, using a secret key.

Step 2. The smart meter transmits the encrypted group session key to all nodes; the nodes decrypt it using the secret key, $b_{m_{i}}$; and then use it for data transmission.

### 4.4. MDMS-Gateway Authentication Phase

Smart meter intra-group authentication ends when session key distribution is completed and MDMS-gateway group authentication may then follow if desired. Unlike the Harn scheme, our scheme features a tree-like hierarchical structure. In this phase, a small group is created by selecting a gateway with smart meters to be communicated with in the MDMS. To do so, the MDMS requests authentication to specific gateway and smart meters, collects authentication information, and sends session information to a subscriber authentication server. The subscriber authentication server registers information about the gateway and MDMS, and the MDMS shares the secret key, k, with the subscriber authentication server. In the subscriber authentication server, session authentication information is transmitted to each MDMS and gateway, and the MDMS and gateway perform authentication and exchange keys. The flow of this phase follows the Park [28] approach and is adapted to the proposed IoT smart metering environment. It can be applied together with the smart meter intra-group authentication scheme proposed above to provide a secure authentication scheme.

Step 1. The MDMS requests access by sending a session identifier SID and its own identifier $ID_{MDMS}$ to the gateway that needs data. The gateway receiving the request forwards the SID and $ID_{MDMS}$ to the smart meters requiring power consumption data.

Step 2. The smart meters that receive the request generate the following message including its $P_{r}$ and sends it to gateway; t is the number of participants in the group that smart meter generates.

(5) $$\left(SID,ID_{s_{1}},P_{r_{1}}\right),\ldots ,\left(SID,ID_{s_{t}},P_{r_{t}}\right)$$

Step 3. The gateway aggregates the $P_{r}$ of each smart meter, generates the following message (6), and transmits it to the MDMS. The MDMS forwards message (6) to the subscriber authentication server.

(6) $$\left(SID,ID_{MDMS},ID_{s_{1}},ID_{s_{2}},\ldots ,ID_{s_{t}},P_{r_{1}},P_{r_{2}},\ldots ,P_{r_{t}}\right)$$

Step 4. The subscriber authentication server selects a random number, *R,* and generates a polynomial $f_{s}\left(x\right)$ using $\left(P_{r_{1}},\ldots ,P_{r_{t}},h_{2}\left(gv∥R\right)\right)$. Then, the subscriber authentication server selects an arbitrary point $P_{s}$ on $f_{s}\left(x\right)$.

Step 5. The subscriber authentication server sends the session authentication value to the MDMS and gateway; i.e., $\left(SID,E_{k}\left(SID,P_{s}\right)\right)$ to the MDMS, and (SID,R) to the gateway.

Step 6. The MDMS decrypts $P_{s}$ from the session authentication value received from the subscriber authentication server and generates polynomial $f_{v}\left(x\right)$ using $\left(P_{r_{1}},\ldots ,P_{r_{t}},P_{s}\right)$. Then, an authentication value $AUTH=h_{1}\left(SID∥ID_{MDMS}∥f_{s}\left(0\right)\right)$ is generated. 

Step 7. The gateway generates $f_{s}\left(x\right)$ using the received session authentication value and $\left(P_{r_{1}},\ldots ,P_{r_{t}},h_{2}\left(P_{s}\right)\right)$. Then, an authentication value $AUTHH^{\prime }=h_{1}\left(SID∥ID_{MDMS}∥f_{s}\left(0\right)\right)$ is generated.

Step 8. The gateway sends $AUTH^{\prime }$ to MDMS, and MDMS authenticates gateway by checking $AUTH=AUTH^{\prime }$. Each gateway and MDMS then generates a session key $SK=h_{1}\left(f_{s}\left(0\right)∥SID\right)$.

## 5. Analysis of Proposed Scheme

### 5.1. Authentication

In this proposed scheme, authentication is divided into smart meter intra-group authentication and MDMS-gateway group authentication. In the smart meter intra-group authentication, the group leader, such as a smart meter, authenticates the node group by performing group authentication from each node. In MDMS-gateway group authentication, the gateway performs authentication with the upper-level MDMS. In addition, the participant nodes send information to the group leader using the identifier and a symmetrical key, receive distributed secret values, and generate tokens using these values. As a correct secret value is generated only when a legitimate token is collected, each genuine participating node can confirm its authenticity. In the MDMS-gateway group authentication, the MDMS selects smart meters through gateways that require authentication and creates a small group to perform authentication.

### 5.2. Preventing Replay Attacks

In the smart meter intra-group authentication, as the attacker does not own a secret key during group authentication, they cannot use a generated token $T_{i}$ to attack. Also, even if the attacker intercepts and retransmits the intermediate value transmitted during group authentication, the attacker cannot acquire the session key because s/he does not own the distributed secret value $f\left(ID_{i}\right)$. 

In the MDMS-gateway group authentication, MDMS generates a session key using a pre-shared master key during the process of identifying and authenticating the identifiers of the gateways through the subscriber authentication server, so that even if an attacker obtains an intermediate MDMS identifier, the session key cannot be acquired. It is designed to be safe against security threats in the smart metering environment described in Section 3.2, and is also designed to prevent replay attacks and to reuse the secret values and key information of each node. For replay attacks, the token $T_{i}$ is an arbitrary value that can only be used in the current session. In order to hide the distributed secret value $c_{i}$, the $P_{i}$ and elliptic curve operations generated by the subscriber authentication server are performed, and the generated token can be verified to be valid only in the current session through the verification value $v_{i}$.

### 5.3. Efficiency

During group authentication, as the number of nodes increases, nodes are authenticated via a single process and one-to-one authentication is lacking. Therefore, the smart meter operates more efficiently than is true of existing schemes. It is possible to increase efficiency further by designing a tree structure accommodating the IoT environment.

**Figure 9:**
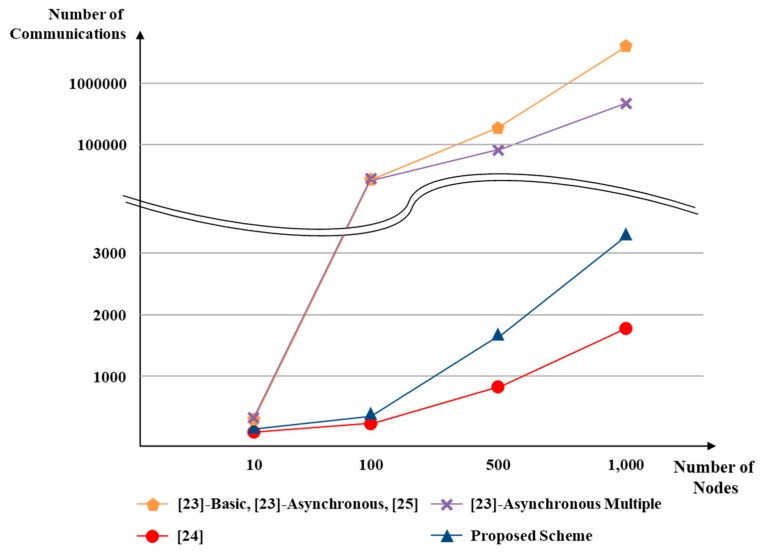
Comparison of GASs by number of communications.

Table 2 compares the existing schemes with the proposed scheme. As a characteristic of the proposed scheme, the group member structure is constructed as a tree form, and the amount of communication in the group authentication phase can be greatly reduced. Figure 9 compares the proposed scheme with the existing group authentication schemes. The total number of nodes participating in the group is *n*, and the number of nodes participating in group authentication is *m*. The proposed scheme does not use one-to-one authentication or a communication structure such as broadcast. Since it uses a 1:N tree type group authentication, it is efficient in terms of the number of communications. In addition, it is designed to prevent replay attacks and to reuse the distributed secret value and key information that each node has provided. This provides greater efficiency than traditional schemes.

### 5.4. Identification of Malicious Participants

As the group leader, such as the smart meter, and the gateway perform authentication using a key managed in collaboration with group participants, a malicious participant cannot engage in any authentication process within the group. Each node receives $b_{m_{i}}$ in the node registration phase, and can generate $v_{i}$ for checking the replay attack, but the unregistered malicious node cannot generate a valid $v_{i}$. Therefore, malicious nodes cannot authenticate through the smart meter. The communication structure is organized in a tree-like manner; the group leader manages group participants and these persons confirm that the group leader is not malicious. 

## 6. Conclusions

We developed a dynamic group authentication and key exchange scheme that operates efficiently in IoT environments. In the intra-group authentication, the group leader performs group authentication by applying a secret sharing threshold, followed by sessional distribution of a symmetrical key to ensure secure communication. As the number of group devices increases, the operation overhead of a group leader traditionally becomes very large. Therefore, our scheme is particularly applicable in IoT environments featuring many group nodes. The scheme prohibits replay attacks and reduces the communication overheads of group leaders. In addition, in the MDMS-gateway group authentication, the data collected from the gateway is designed to be sent to the MDMS securely. From the viewpoint of MDMS, it is possible to dynamically perform authentication by grouping data of the smart meters and gateways to be processed. This provides an efficient and secure IoT smart metering service.

Group authentication is an area of vigorous research. In future, the memory and computation overhead of the group leader will require further reduction, to reduce the amount of computation and communication required.

## Figures and Tables

**Table 1:** System parameters.

Parameter	Explanation
f(x)	A *t* − 1-degree polynomial generating a secret sc $f\left(x\right)=a_{0}+a_{1}x+a_{2}x^{2}+\ldots +a_{t-1}x^{t-1}$
$f_{s}\left(x\right)$	The polynomial generated by the subscriber authentication server
$ID_{m_{i}}/ID_{s_{i}}$	Identifier of meter node/gateway *i*
$f\left(ID_{m_{i}}\right)$	The distributed secret value to each meter node *i*
$h_{r}\left(\cdot \right)$	The random one-way hash function
$h_{1}\left(\cdot \right)$	The mapping hash function of ${\mathbb{Z}}_{p}^{*}\to {\left\{0,1\right\}}^{n}$
$h_{2}\left(\cdot \right)$	The mapping hash function of ${\mathbb{Z}}_{p}^{*}\to \left(x_{1},y_{1}\right)\in f_{s}\left(x\right)$
sc	The secret value generated in the polynomial f(x) $\left(sc=a_{0}\right)$
$SID_{*}$	Identifier of session
p,q	A large prime number (gcd(p,g)=1)
$G_{r}$	A randomly selected point generator on an elliptic curve of order *q*
$P_{s}/P_{sm}$	A randomly selected point on the polynomial by subscriber authentication server/smart meter
X	The smart meter master key
$b_{m_{i}}$	$\mathrm{The}\;\sec\mathrm{ret}\;\mathrm{key}\;\mathrm{for}\;\mathrm{each}\;\mathrm{meter}\;\mathrm{node}\;i\;\mathrm{generated}\;\mathrm{by}\;\mathrm{the}\;\mathrm{smart}\;\mathrm{meter}\;\left(b_{m_{i}}=h\left(X∥ID_{m_{i}}\right)\right)$
k	The symmetric key shared by the subscriber authentication server and MDMS
gv	The secret value shared by the subscriber authentication server and gateway
GSK	$\mathrm{Meter}\;\mathrm{nodes}-\mathrm{gateway}\;\mathrm{group}\;\mathrm{session}\;\mathrm{key}\;\left(GSK=h_{s}\left(P∥X∥sc\right)\right)$
SK	The session key between gateways and MDMS

**Table 2:** Analysis of the scheme.

FeatureComparison	[23]-Basic	[23]-Asynchronous	[23]-Asynchronous Multiple	[24]	[25]	Proposed Scheme
**Base System**	(*t,n*)-threshold	(*t,n*)-threshold	(*t,n*)-threshold DLP	(*t*,*n*)-threshold Bivariate polynomial	(*t*,*n*)-threshold ECDLP	(*t*,*n*)-threshold ECDLP, hash
**Group member participation type**	Broadcasting between nodes	Broadcasting between nodes	Broadcasting between nodes	Participation through group leaders	Broadcasting between nodes	Participation through group leaders
**Group member structure**	Inter-node mesh	Inter-node mesh	Inter-node mesh	Group leader-node Tree type	Inter-node mesh	Group leader-node Tree type
**Prevent replay attack**	X Retransmission when token is disclosed	X Retransmission when token is disclosed	O	O	O	O
**Identification of malicious participants**	Non-verifiable	Non-verifiable	Non-verifiable	Non-verifiable	Verifiable	Verifiable
**Authentication multiple times**	Not provided	Not provided	Reusable keys	Reusable keys	Semi-reusable keys	Reusable keys
**A number of communication during intra-group authentication**	*n* + 2*m*(*n* − 1)	*n* + 2*m*(*n* − 1)	*n* + 2*m*(*n* − 1)	*n* + *m*	*n* + 2*m*(*n* − 1)	*n* + 3*m*

*n*: Number of total nodes in group; *m*: number of nodes participating in group authentication.
